# Occupational exposure to particulate matter from air pollution in the outdoor workplaces in Almaty during the cold season

**DOI:** 10.1371/journal.pone.0227447

**Published:** 2020-01-10

**Authors:** Denis Vinnikov, Zhangir Tulekov, Aizhan Raushanova

**Affiliations:** 1 al-Farabi Kazakh National University, School of Public Health, Almaty, Kazakhstan; 2 National Research Tomsk State University, Tomsk, Russian Federation; 3 Peoples’ Friendship University of Russia (RUDN University), Moscow, Russian Federation; The Ohio State University, UNITED STATES

## Abstract

Background

A large fraction of population in Almaty and other Kazakhstan cities is employed in the outdoor jobs and likely exposed to high levels of particulate matter (PM) during the cold season. The magnitude of such occupational exposure remains unknown; therefore, the aim was to quantify the levels of exposure to PM_10_ in the outdoor workplaces in Almaty in order to guide future interventions of primary prevention.

**Methods:**

Outdoor security non-smoking guards (N = 12) wore TSI DustTrack AM520 aerosol monitors with a 10-μm impactor for 8 hours of outdoor shift. Ten samples (k = 10) from each worker were obtained for the cold season (November-March) from various locations across Almaty. Total sampling time was 57600 minutes. We compared normalized time-weighted average (TWA) concentrations for 8-hour shifts within and between workers using analysis of variance (ANOVA) and assessed compliance with environmental exposure limit (EEL) (0.060 mg/m^3^) via exceedance (γ) and probability of overexposure (θ).

**Results:**

PM_10_ TWA ranged from 0.050 to 2.075 mg/m^3^ with the geometric mean 0.366 and median 0.352 mg/m^3^. PM_10_ TWA distribution was left-skewed with large variation. The fold-range of within-person variability, containing 95% of the exposure concentration (_*w*_*R*_*0*.*95*_) was 13, whereas between-person fold-range (_*b*_*R*_*0*.*95*_) was 3. However, between-person variance exceeded the one within with F-ratio 2.797 (p = 0.003) with statistical power 97% at α = 0.05. Only two of 120 samples had TWA below EEL, yielding γ = 0.995 and θ = 1.

**Conclusions:**

Outdoor workers in polluted cities like Almaty are exposed to very high levels of PM_10_ during the cold season. Urgent action should be taken to regulate such occupational exposure and to raise awareness of workers and employers on hazards associated with it.

## Introduction

Air pollution is a growing public health concern in the majority of countries worldwide, and the burden of pollution with regard to respiratory and cardiovascular morbidity and mortality may be most pronounced in developing countries. With all the green energy revolution and introduction of cleaner technologies, particular matter (PM) air pollution trends are worrisome, and megacities may be at higher risk. Health effects of PM pollution are well-documented and include both short-term [1–4] and long-term effects [5]. In addition, PM_10_ is associated with respiratory effects such as hospitalizations [6], outpatient visits [7] and emergency department admissions for chronic obstructive pulmonary disease (COPD) [8]. Compared to occupational settings, environmental effects are modest, however, millions of people are affected in large cities, and the overall number of victims may be huge, such as in China [9].

The association of PM_10_ environmental pollution with COPD is well-documented [10], whereas the effects of dust and PM_10_ in the occupational context are believed to be greater and the evidence mainly accumulates from dusty workplaces [11,12], which often include construction, production, maintenance or similar jobs. Dusty workplaces are in the focus of the occupational public health initiatives, and various interventions are in place to reduce or mitigate the effects. On the contrary, outdoor workplaces remain out of attention in terms of exposure to PM; however, levels of exposure to PM in them may remain underestimated, should these be located in polluted cities. In Kazakhstan, since the collapse of the Soviet Union and subsequent demolition of central heating infrastructure and the dramatic fall in the economic level of population, also in the suburbs, a large fraction of population in the suburbs uses cheap fossil fuel for heating. This may explain very high concentrations of ambient PM_2.5_ and PM_10_ in Almaty during the cold season, which almost always dramatically exceeds exposure limits [13].

Despite moderate to high correlation between the ambient PM_10_ concentrations and personal exposure [14,15], the former can unlikely serve a surrogate to estimate personal exposure to PM_10_ in the outdoor workplaces, given that the correlation is different for PM_10_ compared to PM_2.5_ [14]. Furthermore, no personal exposure to PM_10_ in the outdoor workplaces has ever been verified in the cities of Kazakhstan. Therefore, the aim of this analysis was to quantify the levels of exposure to PM_10_ in the outdoor *a priori* ‘clean’ workplaces in Almaty with the purpose of guiding future interventions of respiratory primary prevention for such workers.

## Materials and methods

### Study design and participants selection

This exposure assessment study was approved by the Committee on Bioethics of the School of Public Health of al-Farabi Kazakh National University, and every subject signed an informed consent to participate. The city is located at the foot of Ili-Alatau mountain range, and the elevation gradually increases southwards. The population of Almaty is around 2 million inhabitants. Mountains are situated in the southern part of the city, whereas the northern part stretches towards the steppe. Detailed daily real-time monitoring data on PM_2.5_ are available from a number of sensors, set by private or non-governmental bodies and located all over the city [13].

**Fig 1:**
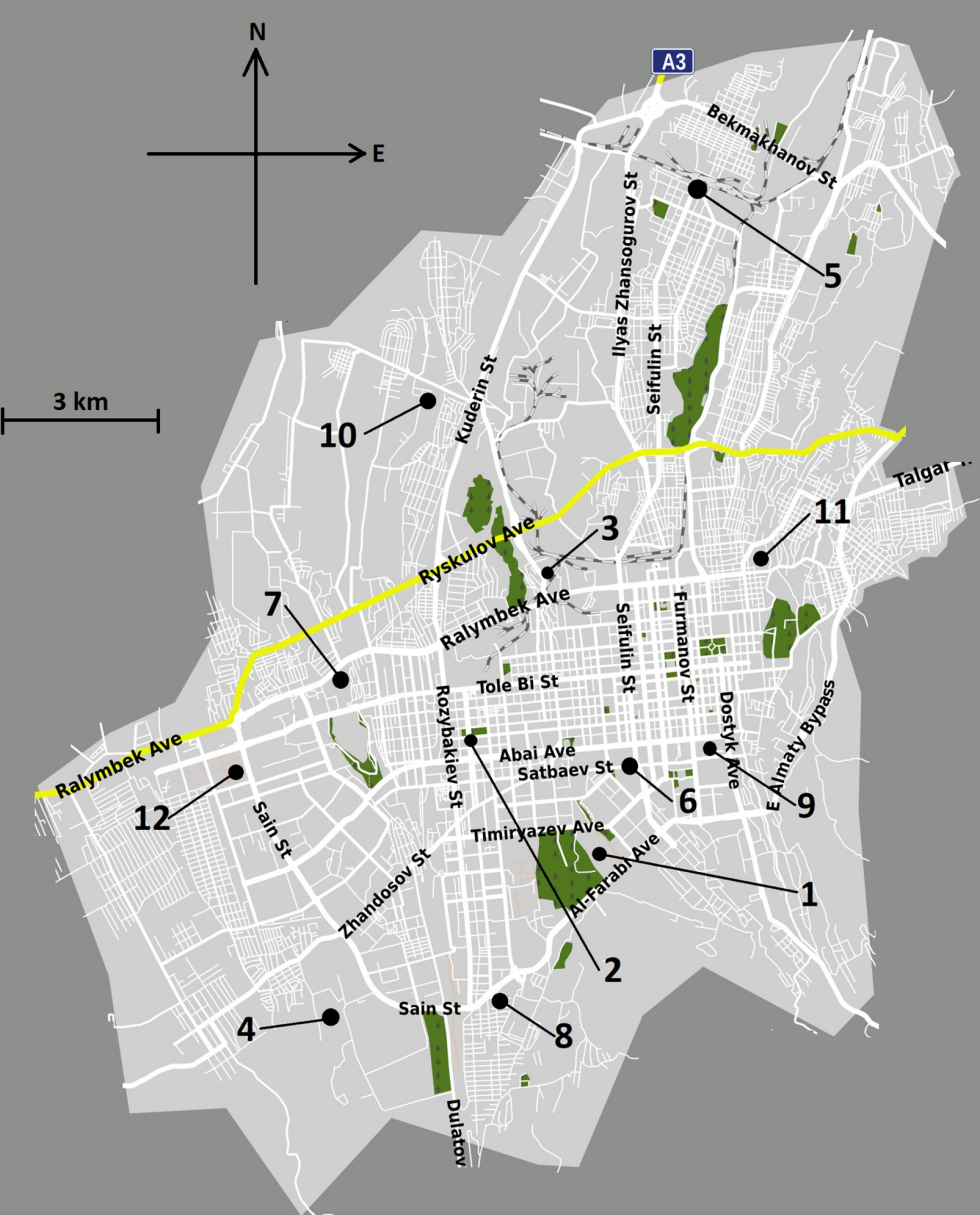
The location of sampling sites in Almaty.

Sampling started in mid-November 2018 and ended in mid-March 2019 and was conducted on an everyday basis. In each worker (= site), ten sampling days were randomly selected from the days of the cold season, and the list of sampling days for each worker (= site) was compiled in the beginning of the study. Cold season in the Southern Kazakhstan starts approximately on November 1 and ends on April 1 (depending on a year), and our sampling covered all four-five months of it. According to the study protocol, sites with private outdoor security personnel, including malls, intercity bus stations and private enterprises were selected for randomization. We compiled the list of such venues in Almaty using online Yandex Maps (www.yandex.ru) (N = 128), whereas eligible sites were selected using randomization code in Microsoft Excel. Twelve sample sites were randomly selected from the list and were equally distributed across the city (Fig 1). Randomization enabled to capture both residential neighborhoods and districts with non-residential infrastructure, such as train station. We obtained 10 samples from each site (= each worker), making the total number of samples 120. The number of sampling sites was informed by approximate cold season duration: in order to obtain 10 samples from each site, we would have 12 sites included, given that the cold season duration is approximately 120 days. We recruited subjects in October 2018, and the relevant demographic details of included subjects are summarized in Table 1. We consider our sample a representative sample of a larger population of outdoor security guards. Outdoor guards were recruited in their workplaces following detailed explanation, and written consent was obtained from the subject along with verbal consent from the supervisor. Should the subject claim daily smoking, we approached the next guard in the same venue until we met a non-smoking employee.

**Table 1:** Demographic characteristics of included subjects.

N	12
Age range, years	19–35
Median age, years	26
Males, N (%)	12 (100)
Kazakh ethnicity, N (%)	11 (100)
Education, N (%)	
Secondary school	5 (42)
High school	5 (42)
College or university	2 (16)
Overall work duration, median years	4

Twelve outdoor security guards, all fit young men aged 19–35 years and working 8-hour outdoors (Table 1) were included. Because smoking is one of the main sources of higher personal exposure compared to ambient sampling, all 12 subjects in this study were non-smokers. We did so to prevent error in measurements from smoking and to ensure that most variation in personal exposure would be due to ambient variation in PM_10_ concentrations. Smoking is only allowed in the designated areas around malls and bus stations; therefore, guards were not exposed to secondhand smoke in the workplace.

### Sampling procedure

We used TSI DustTrak AM520 (USA) personal aerosol monitor, worn on a trouser belt by a security guard for 8 hours. The impactor inlet was connected to a polyvinyl hose with the opposite end attached to a collar with a clip to ensure sampling is done in the breathing zone. We used impactors for PM_10_ for all measurements, whereas device zeroing was done every morning to ensure measurement accuracy. One-minute logging mode was set in all samples, and the overall sampling time for each worker and for each work shift was 480 minutes with a flow rate 1.7 L/min. For each 8-hour sample, we recorded minimum, maximum and an 8-hour time-weighted average (TWA) concentration of PM_10_. Because we logged data every minute, TWA in the current analysis was an arithmetic mean of all measurements logged during 8-hour shift.

### Statistical analysis

The primary outcome in this analysis was 8-hour TWA. PM_10_ TWA was treated as a continuous variable, and we calculated geometric means for each worker and for the entire sample along with the corresponding medians. The analysis is built on TWA concentrations for each test. Shapiro-Wilk was the test of choice to analyze normality of concentrations, and because the data were truncated to the left, all the data analyses were conducted with the normal logarithms of baseline TWAs. Once transformed to normal concentrations, we tested data for variance within (*σ*^*2*^_*wY*_) and between (*σ*^*2*^_*bY*_) persons in the analysis of variance (ANOVA). Because there was one worker for each included site, “between-worker” means both between-worker and between-site variability. To be consistent, we only use “between-worker” term, however, this equals between-site variability also. Total variance of all data (*σ*^*2*^_*Y*_) was the sum of within- and between-person *σ*^*2*^s. We then analyzed relative measures of variability [16], such as (1) the fold-range of between-person variability, containing 95% of the exposure concentration and defined as _*b*_*R*_*0*.*95*_ and calculated as _*b*_*R*_*0*.*95*_ *= exp*^*3*.92*σbY^; and (2) the fold-range of within-person variability, containing 95% of the exposure concentration and defined as _*w*_*R*_*0*.*95*_ and calculated as _*w*_*R*_*0*.*95*_ *= exp*^*3*.92*σbY^. The corresponding fold-range of the total variability was *R*_*0*.*95*_ and equaled the sum of between- and within-person values. _*b*_*R*_*0*.*95*_ and _*w*_*R*_*0*.*95*_ are usually used to compare the magnitude of variability of both variances and with each other. The smoothed trend of PM_10_ concentrations over the entire period was analyzed and plotted using Holt’s liner trend, whereas the smoothing constant was calculated by the program using the mean absolute error (MAE).

Furthermore, we aimed to test the compliance with the existing exposure limits. Because there is no PM_10_ occupational exposure limit (OEL) in Kazakhstan, we attempted to test how many of the overall number of measurements exceeded existing Kazakhstan PM_10_ environmental exposure limit (EEL). This EEL was 0.060 mg/m^3^ for all Kazakhstan cities and was established in 2015. We calculated γ, the exceedance in the group, reflecting the probability of a randomly selected exposure measurement from a group would exceed EEL; and θ, the probability of overexposure, which reflected the probability of a typical person in group to be overexposed. These values were calculated using between- and within-person variances [16] in the formulae:

$$\gamma =1-\Phi \left\{\frac{ln(EEL)-\mu Y}{\sqrt{\sigma^{2}bY-\sigma^{2}wY}}\right\}$$

$$\theta =1-\Phi \left\{\frac{ln(EEL)-\mu Y-\frac{\sigma^{2}wY}{2}}{\sqrt{\sigma^{2}bY}}\right\}$$

In addition, we conducted a longitudinal analysis using mixed effects models and report intraclass correlation coefficient (ρ). All calculations were performed in Excel and in NCSS 12 (Utah, USA), and P-values below 0.05 were considered statistically significant.

## Results

**Fig 2:**
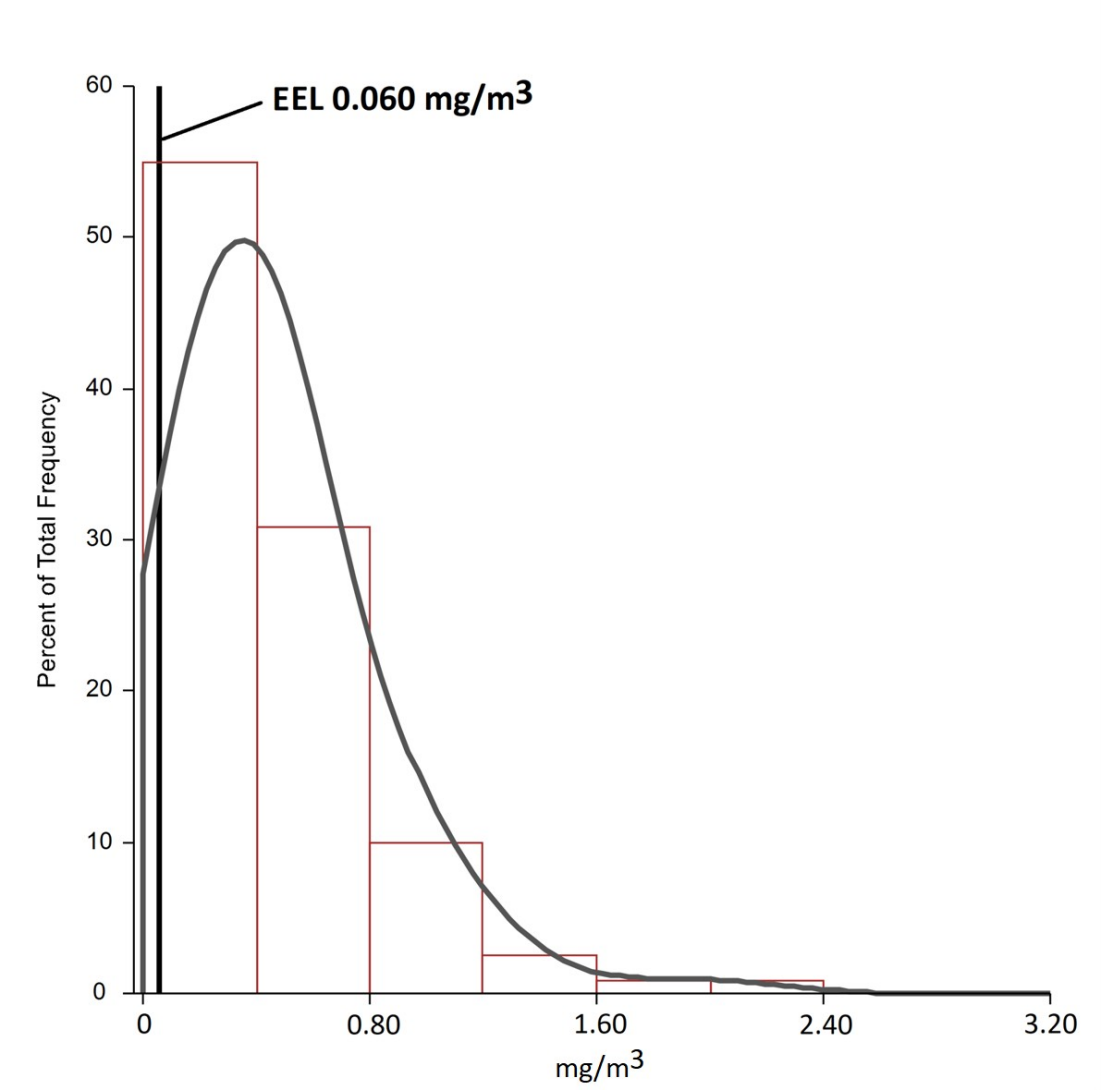
Left-skewed distribution of TWA samples from workers in the study (N = 12, k = 120). Data in the table represent TWAs. EEL–environmental exposure limit.

Of 120 samples collected, the least 1-minute average PM_10_ concentration was 0.006 mg/m^3^, whereas the highest recorded 1-minute average concentration was 5.170 mg/m^3^ (Table 2). PM_10_ TWA ranged from 0.050 to 2.075 mg/m^3^ with the geometric mean 0.366 and median 0.352 mg/m^3^. As expected, PM_10_ TWA distribution was left-skewed (Fig 2) with 95% values below 1.063 mg/m^3^. There was a significant variation in both min and max (from 0.370 to 2.075 mg/m^3^) PM_10_ TWA between workers, whereas PM_10_ TWA median also varied significantly between workers from 0.233 to 0.797 mg/m^3^.

**Table 2:** The means and ranges of recorded PM_10_ concentrations from 12 sampled workers (k = 10 tests from each worker) (sampling started in the middle of November 2018 and ended in the middle of March 2019).

Worker	PM_10_ min, mg/m^3^	PM_10_ max, mg/m^3^	PM_10_ TWA min, mg/m^3^	PM_10_ TWA max, mg/m^3^	PM_10_ TWA geometric mean, mg/m^3^	PM_10_ TWA median, mg/m^3^	Location
1	0.054	1.543	0.181	1.258	0.393	0.307	Botanical garden
2	0.042	2.901	0.192	1.736	0.382	0.261	Gagarin avenue, crossing Shevchenko street
3	0.042	5.170	0.252	2.075	0.739	0.797	Industrial zone on Ryskulov avenue
4	0.015	1.905	0.160	0.831	0.432	0.468	Kamenka district
5	0.037	2.106	0.124	1.234	0.364	0.314	Almaty-I train station
6	0.035	1.559	0.098	0.724	0.284	0.334	Seifullin avenue crossing Satbaev street
7	0.052	1.783	0.144	0.995	0.547	0.586	Rayimbek avenue crossing Otegen batyr street
8	0.010	1.080	0.146	0.833	0.385	0.440	Al-Farabi avenue crossing Gagarin avenue
9	0.010	1.644	0.055	1.053	0.360	0.398	Dostyk avenue crossing Satbaev street
10	0.006	0.995	0.050	0.568	0.199	0.282	Karasu microdistrict
11	0.015	1.692	0.102	0.577	0.307	0.351	Raimbek avenue crossing East Bypass
12	0.038	0.496	0.204	0.370	0.249	0.233	Sayin street crossing Zhubanov street

PM_10_ min and PM_10_ max are 1-minute readings, whereas the remaining data were calculated from series of measurements from each worker. TWA–time-weighted average; EEL–environmental exposure limit

**Fig 3:**
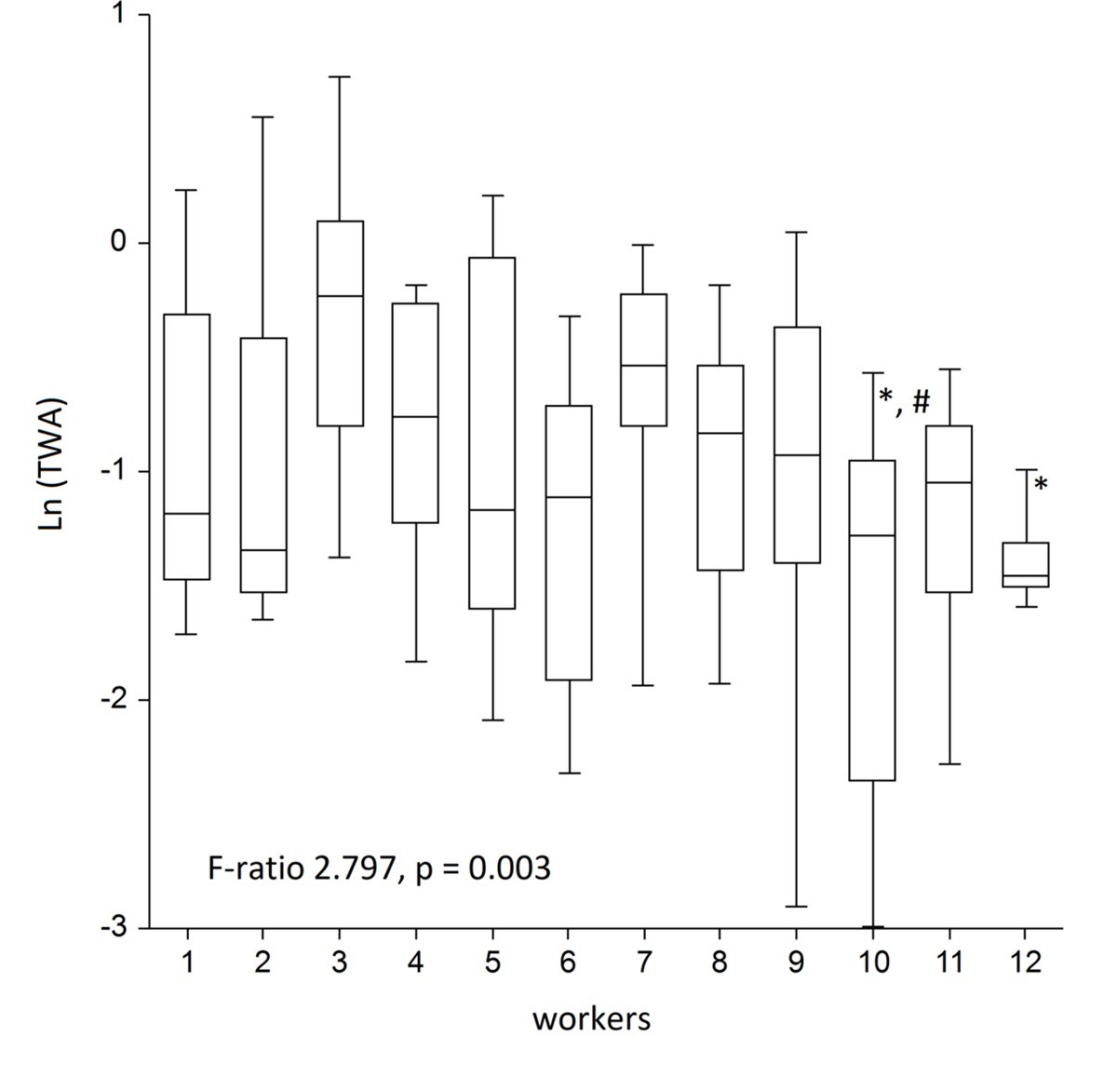
ANOVA of TWA concentrations between workers. Data in the table represent TWAs. *—significant difference from worker 3; #—significant difference from worker 7 (both Tukey-Kramer test). Box with whiskers cover 95% values.

**Fig 4:**
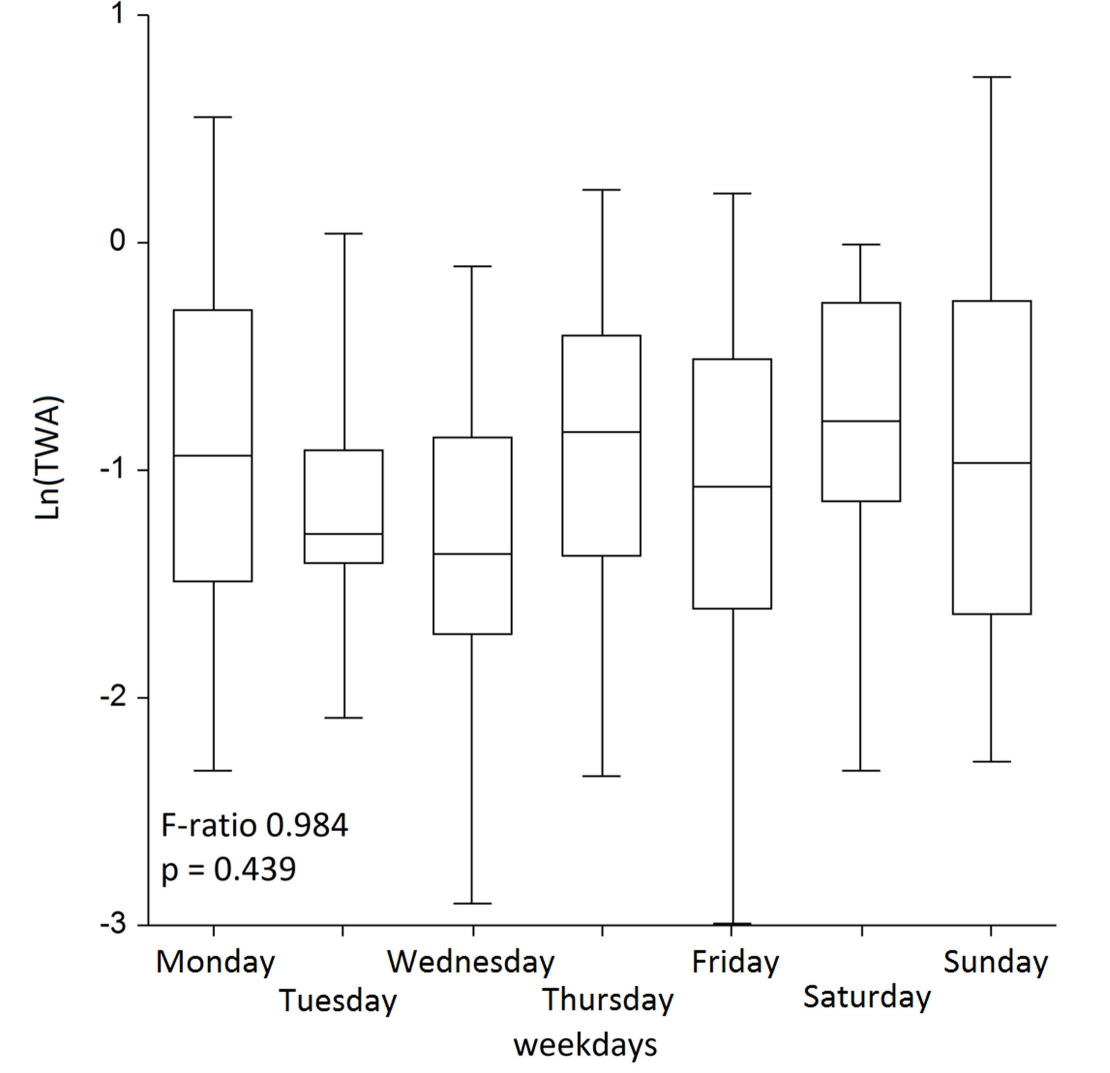
ANOVA of TWA concentrations between weekdays. Data in the table represent TWAs. Box with whiskers cover 95% values.

**Fig 5:**
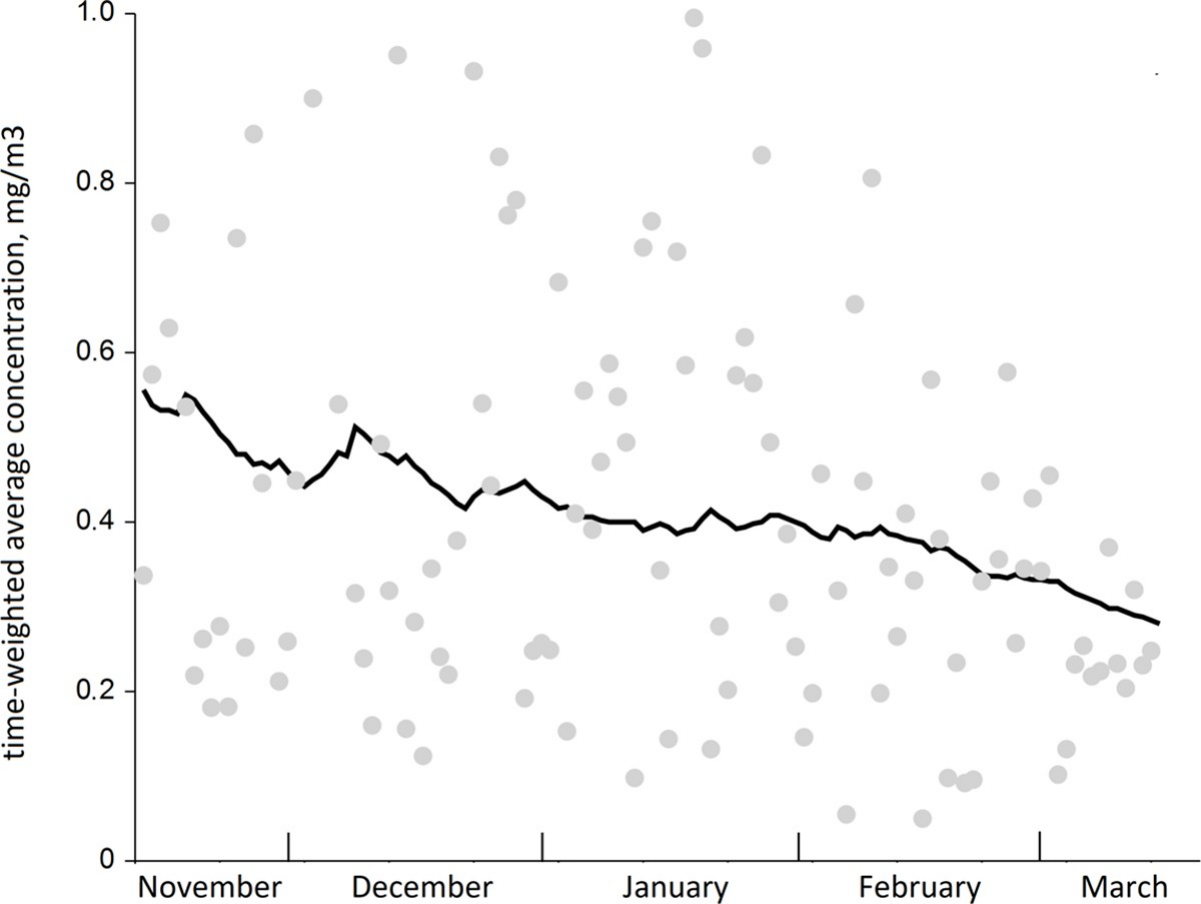
The smoothed trend of TWA concentrations over the entire period from all subjects. Grey dots are actual TWA concentrations for a single day; black line is a smoothed trend.

We tested whether between-worker variability exceeded the one within in ANOVA and found statistically significant F-ratio of 2.797 (p = 0.003), yielding high statistical power of 97% at α = 0.05, indicative of significant differences in TWA concentrations between workers, located in various randomly selected sites across the city (Table 2). Table 3 summarizes within- and between-worker variances. Fig 3 shows the medians with IQR (boxes) with whiskers covering the remaining 45%, thus the total whiskers area showing 95% range of the normalized TWA concentrations. In the *post-hoc* analysis using Tukey-Kramer test, we found that there were significant differences in concentrations of worker 3 from workers 10 and 12; and worker 7 differed from worker 10. In a longitudinal analysis, ρ was 0.30. Fig 3 shows that the overall variance was very large, and that resulted in the fold-range containing 95% of the total variability (*R*_*0*.*95*_*)* 16. Within-person variance made the most contribution to it yielding _*w*_*R*_*0*.*95*_ = 13, leaving 3 for the fold-range of 95% variability of _*b*_*R*_*0*.*95*_. This means that 95% of within-person concentrations’ variability ranged 16-fold, indicative of a very wide range, when compared to between-person variability with only a 3-fold range. Furthermore, we also applied ANOVA to test the hypothesis whether the concentrations could be higher on weekends, since more people would stay at home and burn more fuel for cooking, heating or to relax in their saunas. For this, we grouped concentrations into seven weekdays and found no differences between them (Fig 4). The smoothed trend of TWA concentrations over the entire period is presented in Fig 5.

**Table 3:** Within- and between-worker variances in ANOVA.

Variance	SS	df	MS	F-ratio	p-value	F critical
Between	13.0679	11	1.187991	2.796849	0.003055	1.8783882
Within	45.87413	108	0.42476			
Overall	58.94203	119				

SS–sum of squares; df–degrees of freedom; MS–mean squares

We also tested compliance with Kazakhstan PM_10_ EEL. The majority of TWA concentrations were way higher than the existing EEL of 0.06 mg/m^3^, and only two samples (about 2%) remained below EEL, including one work shift in worker 9 and the remaining one in worker 10. The exceedance of the maximum allowed short-term concentration (0.3 mg/m^3^) was found in all included workers, which confirmed poor air quality in the open workplaces along with low compliance with EEL set by the government. Finally, the probability of a randomly selected exposure measurement from a group would exceed EEL (γ) was 0.995 and the probability of a typical person in group to be overexposed (θ) was 1.0. The corresponding γ for 3 months of winter (December, January and February) only was 0.994.

## Discussion

This is the first presentation of PM_10_ personal exposure data as an alternative to environmental monitoring reports from the outdoor workers during the cold season from the largest city of Kazakhstan with a population of 2 million people. We showed that throughout the entire season of wide fossil fuel use, those working outdoors were exposed to very high concentrations of coarse particles, and within-person variance of exposure may have been huge (_*w*_*R*_*0*.*95*_ = 13), which was probably a function of outdoor temperature, wind direction and other unmeasured determinants. With a probability of overexposure of the ambient TWA EEL 0.06 mg/m^3^, equaling 1.0 in our study, workers in Almaty in these so-called clean non-industrial jobs likely have an elevated risk of PM-related diseases, such as COPD, and action should be taken to reduce fossil fuel combustion for heating and increase population awareness of high risk in such posts.

Kazakhstan is the 9^th^ largest territory in the world with a very developed industry during the time of Soviet Union, which collapsed almost 30 years ago, introducing a colossal shift in population employment from industrial workplaces to sales jobs. A large part of Kazakhstan’s population is now employed in the open markets selling goods, however due to non-industrial nature of such employment such workforce remains out of scope of occupational regulations, including those related to air quality in the workplace. No studies have ever been published in Kazakhstan on health status of the outdoor workers, despite the fact that air quality continues to deteriorate. The reasons for devastating air quality in Almaty and Astana, the largest Kazakhstan cities, are likely of economic origin, because the population cannot afford natural gas for heating and has to use more affordable fossil fuel, including dung, to heat the buildings in the suburbs. In addition, Astana, the country capital, has never had centralized natural gas supply, whereas in Almaty, coarse PM air pollution during the cold season likely originates from two sources, including Central Heat and Power Station, providing centralized service of hot water and heating for the city and powered by coal, and burning fossil fuel in the suburbs.

No high-quality research is yet available on the chemical composition verifying the origin of PM pollution, however, burning fossil fuel in the suburbs may play the greatest role in the spiking concentrations of PM during the cold season, since ambient PM_2.5_ concentrations remain below EEL in summer, when Heat and Power Station is still running. Independent PM_2.5_ monitoring with low-cost sensors by www.airkaz.org confirms contrasting levels of PM in the city during the cold season compared to clean summer seasons; however, data interpretation is limited to the assumptions associated with the use of low-cost sensors. No data is available from Kazakhstan cities as to whether highly polluted days lead to an increase in the number of admissions to the outpatient facilities and hospitals with asthma attacks, rhinitis or respiratory symptoms; however, extrapolating studies outcomes from elsewhere [17], days with higher PM_10_ concentrations will put a greater burden on the primary healthcare system due to an increasing number of admissions. Moreover, we found no information in the literature on the awareness of Kazakhstan population on the occupational hazards of polluted air in the outdoor workers, which hampers mitigation activities, since the risk in outdoor security guards or market sellers is underestimated.

Two samples out of almost 120 samples in our study were still below ambient EEL, contrasting with other very high PM_10_ concentrations (maximum recorded level 5.170 mg/m^3^), indicative of probably multiple sources of huge variance, including wind direction. Independent of PM sources in Almaty, personal exposure in the outdoor workplaces was so high, that required immediate action. Direct comparison of the mean concentrations in our study with reports from other countries, albeit using different study designs and exposure assessment techniques, shows that air pollution in Almaty may be as severe as in other most polluted cities around the world. In published reports, outdoor concentrations are more reported than personal exposure levels, but even their comparison prompts great variability. Thus, concentrations range from 0.300 mg/m^3^ in the buses [18], 0.218 mg/m^3^ in metro [19], to much lower readings even close to mine dumps in South Africa (0.016 mg/m^3^ [20]) and did not exceed 0.044 mg/m^3^ in the mining area of Australia [21] or 0.073 mg/m^3^ in the cold period in Athens [22]. In addition, personal PM_10_ concentration in a polluted city Padova did not exceed 0.334 mg/m^3^ in winter, whereas the median was 0.067 mg/m^3^ [23], much lower than in Almaty.

Although no clinical examination was planned in this analysis, data extrapolated from other cohort and large studies will explain enormous health effects of Almaty pollution in the city population, given almost 2 million people are affected. Those health effects are widely supported by molecular studies, which all conclude on the inflammation associated with the inhalation of PM [24]. Studies outcomes are more consistent in cardiovascular mortality and all-cause mortality, when PM_2.5_ is considered as opposed to PM_10_ [25]. However, respiratory effects of PM_10_ exposure are well-documented, and the pooled change of FEV_1_ as an example for every 0.01 mg/m^3^ increase was -3.38 ml [26]. This means that for our population exposed in the workplace, a 0.3 mg/m^3^ increase from 0.03 to 0.33 mg/m^3^ will result in a 30*3.38 = 101 ml decrease of FEV_1_. Coupled with other effects, including increased risk of hospitalizations, outpatient visits and emergency department admissions for COPD, pollution levels typical for Almaty in cold season have a devastating effect on respiratory health.

We consider the analysis of personal exposure data as opposed to fixed environmental monitoring data on air pollution an advantage of our report. In many presentations elsewhere they analyzed PM satellite data, which may not truly reflect population exposure, given unknown time spent indoors/outdoors, smoking pattern, level of physical activity, buildings density in the city and other unmeasured confounding. Although the correlation between fixed-site measurements and personal mean concentrations for PM_10_ was acceptable (r = 0.58) [15], personal measurements are advantageous, but require more resources and may yield variability in concentrations. Another strength of this analysis is the overall long sampling time, 960 hours. Finally, we were able to characterize the exceedance and probability of overexposure in addition to exposure variability, and that provided deeper insight into the exposure portrait of outdoor workers. Limitations of this analysis should also be noted. We did not aim to compare our data with the clean summer season, because summer PM concentrations are often below EEL, as reported by the alternative projects, such as www.airkaz.kz. Another limitation is the use of only one marker of air pollution, PM_10_, while comparison with gases, such as NOx or ozone, could shed more light onto the chemical composition of Almaty air pollution and even the origin of it. Thirdly, the number of subjects in the current analysis may be small to allow wide extrapolation of our findings, despite sufficient number of measurements per subject. We also note that the influence of air and flow on the air pollution levels could not be ascertained in the current presentation, because this information was not available. Finally, we had to compare 8-hour TWA with 24-hour environmental exposure limits.

Our study findings have distinct policy implications. First and foremost, “clean” outdoor workplaces are not clean in polluted cities like Almaty during the cold season because of fossil fuel use for heating, and all outdoor workers are likely overexposed. PM concentrations may not be as high as they traditionally report from industrial worksites, however, the levels may be significant to increase the risk of respiratory and cardiovascular diseases, such as COPD. Therefore, the conventional list of occupations associated with COPD should not only be confined to vapors, gases, dusts and fumes (VGDF) posts, but also include outdoor jobs in polluted cities. Secondly, given high fraction of population is employed in sales in the outdoor markets like in Kazakhstan, the population at risk is not limited to outdoor security guards, but also includes outdoor market sellers, the number of which is hard to ascertain, but likely exceeds tens of thousands of people in Almaty only. At present, the overall ratio of exposed population is impossible to ascertain, since many jobs in security, sales in the markets and other similar posts remain informal. In addition, informal labor is not under regulation in Kazakhstan. Finally, high exposure levels in these posts necessitates more attention to regulation, and the absence of PM_10_ occupational exposure limit is one of the examples of serious gaps in environmental and occupational surveillance legislation. Effort should be made to raise awareness of such vulnerable population groups on the risks associated with their employment and promote shift to work indoors during the cold season. This will not eliminate hazards associated with PM_10_ pollution due to some infiltration indoors, but may reduce risk, because PM concentrations indoors may be lower.

## Conclusions

In conclusion, this first presentation of PM_10_ concentrations in the outdoor workplaces during the cold season in Almaty, the largest city in Kazakhstan, confirmed very high levels of exposure with great variability when compared to environmental exposure limits set by the government. Outdoor workers in Almaty during the cold season are at high risk of respiratory diseases, and the risk is significantly underestimated. The population may be unaware of hazards of working full day shift outdoors during the cold season, and action should be taken to combat air pollution in Kazakhstan.
